# Introduction to this special issue “Breast Cancer Metastasis”

**DOI:** 10.20517/2394-4722.2020.01

**Published:** 2020-02-14

**Authors:** William P. Schiemann

**Affiliations:** Case Comprehensive Cancer Center, Case Western Reserve University, Cleveland, OH 44106, USA.

Breast cancer remains the most common malignancy and most frequent cause of cancer-related death in women, a devasting reality that annually claims more than 600,000 lives across the globe^[1]^. The vast majority of deaths due to breast cancer are attributed to metastasis and its associated relapse^[2,3]^, which typically transpires in patients ~5-20 years after their initial diagnosis^[4]^. Although metastasis is the most lethal characteristic of breast cancer, our understanding of the molecular mechanisms that govern this event remains incomplete, a stark reality reinforced by the finding that diagnosis of distant-stage disease has remained unchanged over the course of the last two decades^[5]^.

The metastatic cascade is a highly complex and inefficient process subject to regulation by a host of intrinsic and extrinsic cellular mechanisms^[6]^. Breast cancers most frequently disseminate to the brain, bone, and liver^[7]^, doing so by undergoing a distinct sequence of events that involve: (1) local invasion through the epithelial basement membrane and migration through stromal connective tissue; (2) intravasation into blood or lymphatic vessels to facilitate dissemination; and (3) extravasation and infiltration into tissue parenchyma, followed by eventual colonization of secondary organ sites^[6,8,9]^. Importantly, the rapid development of powerful new technologies and models to study breast cancer metastasis has greatly expanded our appreciation of the complexities associated with this last frontier of cancer biology. This Special Issue on Breast Cancer Metastasis is a compendium of 17 review articles, 13 original articles, and 3 case reports that collectively cover a wide spectrum of topics associated with the development and treatment of breast cancer metastases. Among the review topics presented herein are thought-provoking discussions on the potential impact of estrogen disrupting agents^[10]^ and *ESR1* mutations to engage the metastatic cascade^[11]^, as well as eloquent analyses of how fluidity^[12]^ and plasticity^[13]^ within the hierarchy of normal and malignant stems cells drives breast cancer development and metastatic progression. Similarly, Robinson *et al.*^[14]^ provide an elegant perspective on how alternations in telomere maintenance mechanisms influence the metastatic evolution of breast cancer stem cells. Likewise, thoughtful discussions on the regulation of epithelial-mesenchymal transition (EMT) programs and carcinoma plasticity also extend into new arenas of: (1) epigenetic modification of histones by lysine-specific demethylase (LSD1 or KDM1A^[15]^); (2) spatiotemporal control of the translatome coupled to EMT by aberrant expression and activation of hnRNP E1^[16]^; and (3) mRNA splicing factors and their essential contribution to the acquisition of EMT and metastatic phenotypes^[17]^. Along these lines, Maisel and Schroeder^[18]^ present an intriguing and comprehensive discourse on how defects in retrograde trafficking of cytokine and growth factor receptors contribute to the acquisition of metastatic phenotypes in breast cancer.

Science and medicine currently lack the knowledge to understand how disseminated breast cancers escape clinical detection by remaining dormant for years before reemerging as chemoresistant and incurable secondary tumors^[19,20]^. Indeed, the mysteries of metastatic dormancy have been identified as one of the ten most critical research gaps and translational priorities needed to be solved to alleviate breast cancer^[21]^. Herein, the paradoxical role of autophagy in both suppressing and promoting breast cancer development and metastatic progression is discussed, particularly its importance in maintaining dormancy-associated phenotypes in disseminated breast cancer cells^[22]^. Gooding *et al.*^[23]^ highlight the far-reaching impact of the noncoding genome and the long noncoding RNA BORG in regulating breast cancer metastasis, chemoresistance, and disease recurrence during metastatic dormancy. Not to be forgotten are the essential functions of the tumor microenvironment in regulating breast cancer biology and metastatic progression^[24]^. Indeed, the therapeutic potential of disrupting breast cancer cell communication with stromal factors and cryptic peptides is presented^[25]^, as are intriguing discussions of how: (1) involution in the postpartum mammary gland engenders a pro-metastatic microenvironment reminiscent of those found in their tumorigenic counterparts^[26]^; and (2) stress and inflammatory signals disrupt neuronal circuitry, thereby contributing to brain metastasis by breast cancers^[27]^.

This Special Issue also considers the translational and clinical aspects of therapeutic targeting metastatic breast cancers. Indeed, Kamal *et al.*^[28]^ present a comprehensive review comparing active *vs.* passive treatment strategies to alleviate breast cancer metastasis to the brain, while dos Santos *et al.*^[29]^ offer an equally far-reaching review on the molecular mechanisms underlying photodynamic therapy and its potential to treat metastatic disease. Additionally, the clinical utility of monitoring circulating tumor cellsas a measure of disease progression and therapeutic effectiveness is also discussed^[30]^.

Finally, it is our hope that these timely and topical reviews will prove to be intellectually stimulating and highly thought-provoking. Likewise, we invite you to explore the 13 original articles and 3 case reports, which dovetail topically with the aforementioned reviews.
